# Supplementary material for: Interaction of TLR4 and TLR8 in the Innate Immune Response against Mycobacterium Tuberculosis
Source: Int J Mol Sci. 2021 Feb 4;22(4):1560. doi: 10.3390/ijms22041560 (PMC7913854; doi:10.3390/ijms22041560)
Supplement: Supplementary file 1 [file ijms-22-01560-s001.pdf]

Supplementary Data

Tables

Table S1: Epidemiological characteristics of the Indian cohort consisting of TB patients and controls

Variables		Controls	Primary TB cases			Relapse cases
			All patients	PTB	EPTB	
		301 (100)	346 (100)	224 (100)	121 (100)	95 (100)
**gender (N=767)***	female	143 (50.89)	204 (58.96)	124 (55.36)	80(66.12)	47 (49.47)
	Male	138 (49.11)	142 (41.04)	100 (44.64)	41 (33.88)	48 (50.53)
**BCG (N=637)****	Yes	223 (82.90)	148 (48.84)	96 (51.61)	52 (44.44)	24 (25.26)
**BMI (N=648)*****	mean[SD]	24.30 [0.30]	17.87 [0.24]	16.56 [0.22]	19.94 [0.43]	16.49 [2.38]
**Age (N=668)******	mean[SD]	32.90 [059]	25.51 [0.60]	25.96 [0.82]	24.79 [0.86]	30.63 [11.08]
**TLR8-Met1Val**	A/AA/AG	139 (47.60)	199 (58.70)	124 (56.62)	75 (63.03)	68 (72.34)
	G/GG	153 (52.40)	140 (41.30)	95 (43.38)	44 (36.97)	26 (27.66)
**TLR4-Thr399Ile (N=717)**	CT/CC	223 (76.32)	229 (68.56)	146 (66.97)	82 (71.30)	52 (61.18)
	TT	68 (23.37)	105 (31.44)	72 (33.03)	33 (28.70)	33 (38.82)
**TLR4-Asp299Gly (N=654)**	AA	190 (72.52)	201 (58.09)	122 (65.59)	79 (69.30)	56 (58.95)
	AG/GG	72 (27.48)	100 (28.90)	64 (34.41)	35 (30.70)	36 (37.89)

* gender differed significantly between controls and primary TB (χ^2=4.08, p=0.043), but not between primary TB and relapse cases (χ^2=2.73, p=0.098).

**BCG vaccination status differed significantly between controls and primary TB (χ^2=72.51, p<0.001), but not between primary TB and relapse cases (χ^2=3.06, p=0.080).

***mean BMI differed significantly between controls and primary TB (t(571)=17.08, p<0.001), as well as between primary TB and relapse cases (t(376)=9.93, p<0.001)

****mean age differed significantly between controls and primary TB ($t(572)=8.72$, $p<0.001$), as well as between primary TB and relapse cases ($t(396)=4.07$, $p<0.001$)

Table S2. Comparison of German and Indian Healthy individuals regarding TLR-4-Thr399Ile (C>T) and TLR-4-Asp299Gly (A>G) allele status.

**TLR SNPs**		**TLR4-Asp299Gly_ TLR4-Thr399Ile***			
**Haplotype**		A_C	G_T	G_C	A_T
**Indian***	**Frequency**	0.813	0.095	0.058	0.034
	**D**	0.0748			
	**D′**	0.687			
	**r**	0.622			
	**Cosegregation [%]**	73			
**German****	**Frequency**	0.9404	0.0518	-	0.0078
	**D**	0.049			
	**D′**	0.999			
	**r**	0.928			
	**Cosegregation [%]**	98			

* Indian healthy subjects consisted of individuals from the TB cohort control group recruited Hyderabad, Andra Pradesh

** German healthy subjects consisted of an internal Institute´s control cohort in Berlin

*** Hardy-Weinberg-Equilibrium for both TLR4-Asp299Gly (P=0.140) and TLR4-Thr399Ile (p=0.590) were not significant. Statistic were calculated using SNPStats (https://www.snpstats.net/start.htm?)

Table S3. Model for the impact of TLR4-Thr399Ile on being a primary TB case, compared to healthy controls.

Variable	OR [95%CI]**	Std Err	z	p
**TLR4-399T***	0.64 [0.42-0.95]	0.13	-2.19	0.028
**gender**	0.69 [0.48-0.99]	0.13	-1.97	0.048
**age**	0.93 [0.91-0.95]	0.01	-7.64	0.947
**constant**	15.56 [7.88-30.71]	5.8	7.91	<0.001

* Likelihood Ratio comparing model with and without TLR4-399T: chi2(1)=4.88, p=0.027

** ORs calculated with Wald's test.

Table S4. Model for the impact of the interaction of TLR8-M1V and BCG-status on being a primary TB case, compared to healthy controls.

Variable	OR [95%CI]**	Std Err	z	p
**TLR8-1G**	1.16 [0.57-2.33]	0.41	0.4	0.687
**BCG**	0.38 [0.22-0.65]	0.15	-3.5	<0.001
**TLR8-1G x BCG***	0.47 [0.20-1.07]	0.2	-1.79	0.073
**gender**	0.88 [0.60-1.29]	0.17	-0.66	0.507
**age**	0.94 [0.92-0.96]	0.01	-6.51	<0.001
**constant**	17.76 [8.79-35.89]	6.38	8.01	<0.001

*Likelihood Ratio comparing model with and without interaction term: chi2(1)=3.26, p=0.071

** ORs calculated with Wald's test.

Table S5. Model for the impact of TLR8-M1V on being a relapse, compared to primary TB cases.

Variable	OR [95%CI]**	Std Err	z	p
**TLR8-1G***	0.49 [0.26-0.90]	0.15	-2.29	0.022
**BCG**	0.46 [0.25-0.83]	0.14	-2.56	0.011
**gender**	2.19 [1.20-3.99)	0.67	2.55	0.011
**age**	1.04 [1.02-1.07]	0.01	3.29	0.001
**constant**	0.08 [0.38-0.18]	0.03	-6.26	<0.001

* Likelihood Ratio comparing model with and without TLR8-1G : chi2(1)=5.53, p=0.019

** ORs calculated with Wald's test.

Table S6. Primers used for Light Cycler Assays (Roche).

Gene	Function	Primer
**TLR8 M1V (BioTez)**	Forward	TCAGGAAGTTAGCCAGTTTCTC
	Backward	CCTGCATTTACAGTTGTTTCGAT
	Sensor	AAATAGAAGTGGCTTACCACGTTTCTGT-FITC
	Anchor	Cy5-TTCTAATTTTTCATTCCGTAACTTGCAGCAGCGCA
**TLR4-Thr399Ile (TIB Molbiol)**	Forward	ATTTAAAGAAATTAGGCTTCATAAGCT
	Backward	CCAAGAAGTTTGAACTCATGGTAA
	Sensor	LC640-ATTTTGGGACAACCAGCCTAAAGTAT
	Anchor	CTTGAGTTTCAAAGGTTGCTGTTCTCAAAGT-FL
**TLR4-Asp299Gly (TIB Molbiol)**	Forward	ATTTAAAGAAATTAGGCTTCATAAGCT
	Backward	CCAAGAAGTTTGAACTCATGGTAA
	Sensor	CTACTACCTCGATGATATTATTGA-CTTATT-FL
	Anchor	LC640-AATTGTTTGACAAATGTTTCTTCATTTTCC-PH

Table S7. Primers for Mutagenesis with QuikChange II XL Site-Directed Mutagenesis Kit (Agilent Genes).

Gene	Function	Primer
**TLR8 M1V (BioTez)**	Forward	5'GAGATCACCGGTCACCGTGGAAAACATGTTC3'
	Reverse	5'GGAACATGTTTTCCACGGTGACCGGTGATCTC3'
**TLR4-Thr399Ile (TIB Molbiol)**	Forward	5'GCATACTTAGACTACTACCTCGATGG-TATTATTGACTTATTTAATTGTTTGA3
	Reverse	5'TCAAACAATTAAATAAGTCAATAATACCATCGAGGTAGTAG-TCTAAGTATGC3'
**TLR4-Asp299Gly (TIB Molbiol)**	Forward	5'AAATACTTTAGGCTGATTGTCCCAAAATCACTTTGA-GAACAGCA3
	Reverse	5'TGCTGTTCTCAAAGTGATTTTGG-GACAATCAGCCTAAAGTATTT3'

Figure S1. TLR4 peptide HCD Spectrum

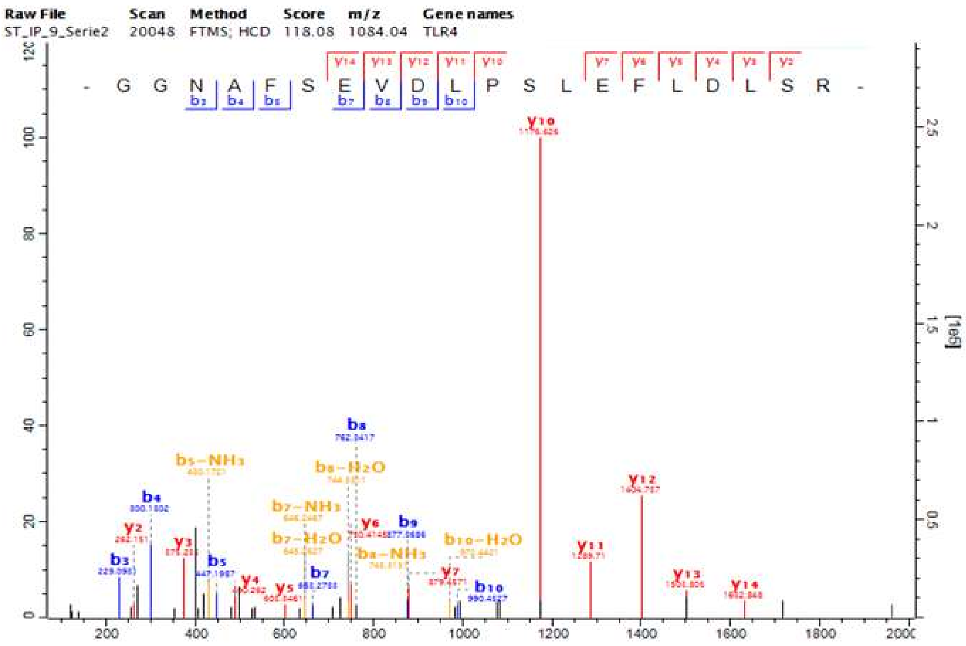

Figure S1: TLR4 peptide HCD Spectrum. TLR4 peptide HCD Spectrum. Successful identification of TLR4 peptide in a TL8/4 co-IP sample. Y and b are the ions generated after tryptic digestion (MS2 spectra). Y-axis: rel. intensity and x-axis: mass over charge ratio

Figure S2: Co-immunoprecipitation of TLR4 and -8 with MD2 and CD14

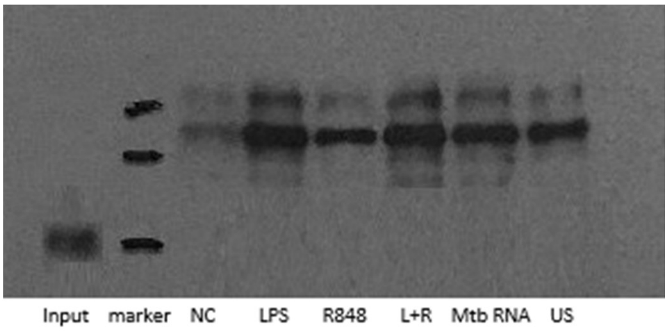

Figure S2: Co-immunoprecipitation of TLR4 and -8 with MD2 and CD14. HEK293 cells were transiently transfected with hTLR8HA and TLR4 399C-mCherry-myc 8 with MD2 and CD14 followed by LPS (10ng/ml), R848 (2µg/ml), LPS+R848 (L+R), Mtb RNA (1µg/ml) stimulation as shown above for 2 h. IP was performed with anti-HA-antibody and blot with anti-TLR4-antibody. Input: pre-IP sample, Nc: negative control. US: unstimulated.

Figure S3: Co-immunoprecipitation of TLR7 and -4

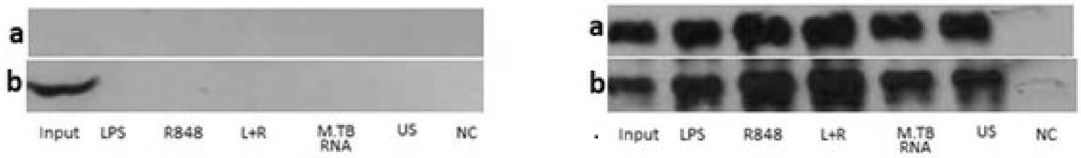

Figure S3: Co-immunoprecipitation of TLR7 and -4. HEK293 cells were transiently transfected with **a.** hTLR7FLAG, **b.** hTLR7FLAG + TLR4 399C-mCherry-myc followed by stimulation as shown above for 2 h. The left panel shows immunoprecipitation with anti-FLAG antibody and -blot with anti-TLR4 antibody, while the right panel shows immunoprecipitation and - blot with anti-FLAG antibody.

Figure S4: Co-immunoprecipitation of TLR8 and Rhesus-TLR4 or C.atys-TLR4

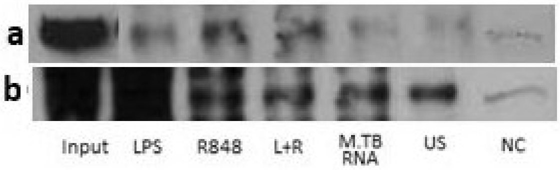

Figure S4: Co-immunoprecipitation of TLR8 and Rhesus-TLR4 or C.atys-TLR4. HEK293 cells are transiently transfected with **a.** RhesusTLR4-FLAG+hTLR8HA+UNC93B1, **b.** C.atysTLR4-FLAG+hTLR8HA+UNC93B1, followed by stimulation as shown above for 2 h.

The left panel shows immunoprecipitation with anti-HA antibody and –blot with anti-FLAG antibody

Figure S5. Co-transfection of TLR4, - 8 and MD2 and CD14

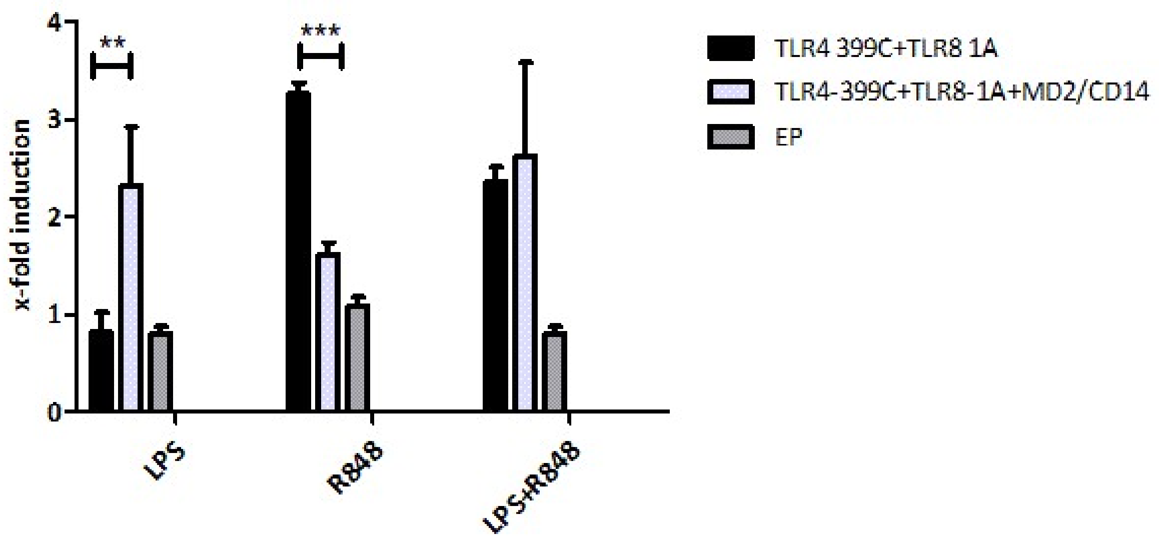

Figure S5: Co-transfection of TLR4, -8 and MD2/CD14. HEK293 blue null 1 cells were overexpressed with hTLR4 and –8 with or without addition of MD2 and CD14 as indicated or empty plasmid (EP), stimulated for 2h with LPS (10ng/ml), R848 (2µg/ml) or LPS+R848 and assessed for NF-κB via SEAP reporter gene assay. Adding MD2 and CD14 increased LPS responsiveness ($t=4.29$, $p=0.006$), but decreased R848 responsiveness ($t=17.26$, $p<0.001$).

Figure S6. Co-transfection of TLR4 and -8 and respective variant forms

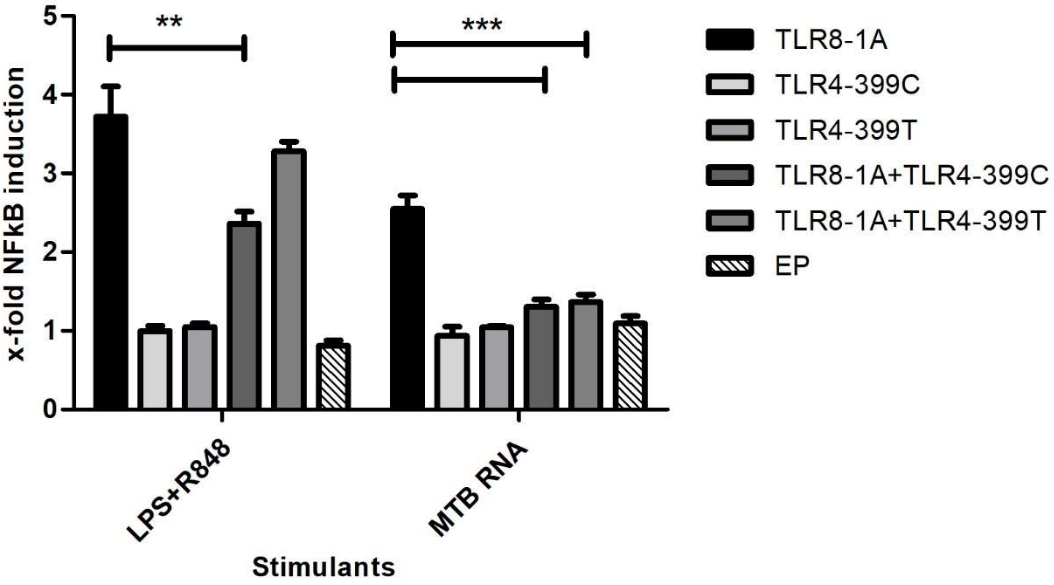

Figure S6: Co-transfection of TLR8 with variants of TLR4. HEK293 blue null 1 cells were overexpressed with hTLR8 and variant plasmids of hTLR4 or empty plasmid (EP), stimulated with LPS+R848 (10ng/ml+2µg/ml for 2h) and Mtb RNA (1µg/ml for 16h) and assessed for NF-κB via SEAP reporter gene assay. For LPS+R848, when adding to TLR8-1A TLR4-399C, NFκB-levels were significantly lower (p=0.05) compared to only TLR8-1A. In combination with TLR4-399T, the difference upon stimulation with LPS+R848 was not significant (p=0.134). For Mtb RNA, both adding TLR4-399C and TLR4-399T were significantly decreasing NFκB-induction (p<0.001) compared to transfection with only TLR8-1A.

Figure S7: TLR8 signalling inhibition with Bafilomycin or psiTLR8 in THP monocyte-derived macrophages cells

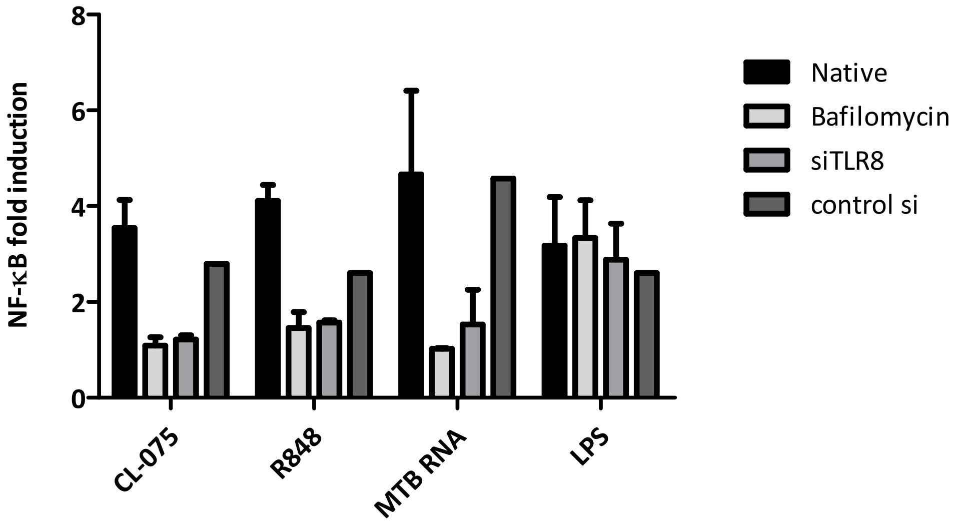

Figure S7: Endosomal signalling adaptor protein inhibition with Bafilomycin or siTLR8 in THP monocyte-derived macrophages cells. THP monocyte-derived macrophages were stimulated with CLO-075 (2 µg/ml), R848 (5 µg/ml), Mtb RNA (5µg/ml complexed with Lyovec) or LPS (10 ng/ml) with or without pre-treatment with bafilomycin or siTLR8. TLR8-ligand stimulation significantly decreased after bafilomycin treatment and siTLR8.

Figure S8: LPS contamination check

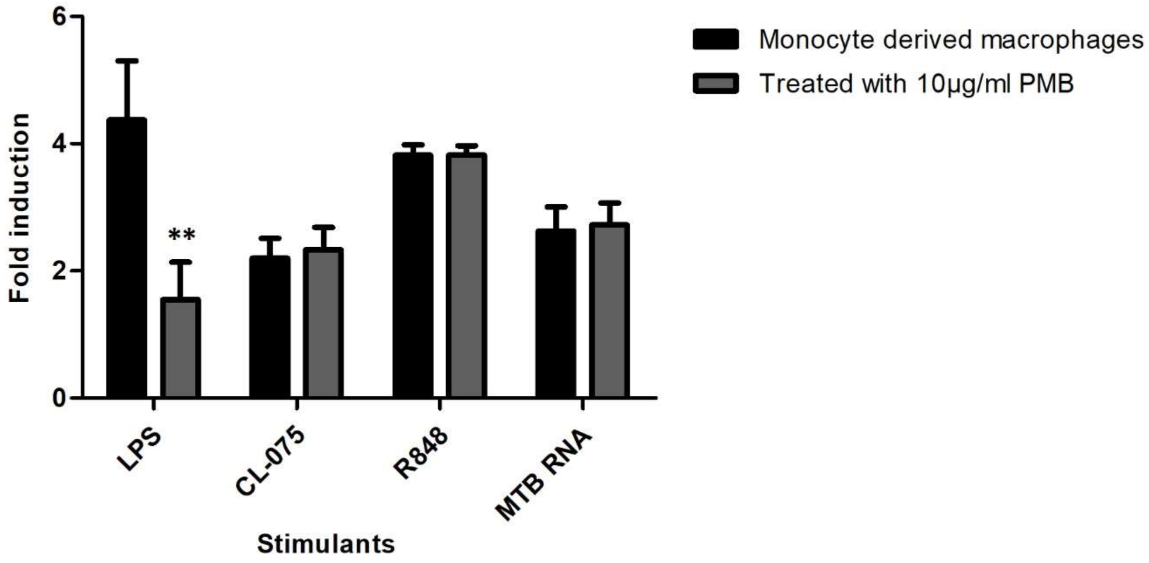

Figure S8: LPS contamination check. THP NFKB monocyte-derived macrophages were treated with PMB (10 µg/ml) and stimulated with CLO-075 (2 µg/ml), R848 (5 µg/ml), Mtb RNA (5µg/ml complexed with Lyovec) or LPS (10 ng/ml). LPS stimulation index significantly reduced in cells treated with PMB ($p<0.01$), all other stimulants did not show any reduction indicating there is no LPS contamination.
